# Study on the Relationship Between the Expression of B Cell Mature Antigen and the Classification, Stage, and Prognostic Factors of Multiple Myeloma

**DOI:** 10.3389/fimmu.2021.724411

**Published:** 2021-11-18

**Authors:** Tiantian Ma, Jing Shi, Yuxia Xiao, Tianyue Bian, Jincheng Wang, Lingyun Hui, Mengchang Wang, Huasheng Liu

**Affiliations:** ^1^ Department of Hematology, The First Affiliated Hospital of Xi’an Jiaotong University, Xi’an, China; ^2^ Department of Hematology, Xi’an No. 3 Hospital, the Affiliated Hospital of Northwest University, Xi’an, China; ^3^ Department of Respiratory and Endocrinology, The Second Affiliated Hospital of Xi’an Jiaotong University, Xi’an, China; ^4^ Clinical Laboratory, The First Affiliated Hospital of Xi’an Jiaotong University, Xi’an, China; ^5^ Biobank, The First Affiliated Hospital of Xi’an Jiaotong University, Xi’an, China

**Keywords:** B cell mature antigen, BCMA, multiple myeloma, MM, chimeric antigen receptor T cells therapy, CAR-T

## Abstract

The expression level of BCMA in bone marrow of 54 MM patients was detected in this study to explore the relationship between the BCMA expression and the classification, stage, and prognostic factors of MM. The BCMA expression level of the stable group and remission group was lower than that of the newly diagnosed group and relapse group (P=0.001). There was no significant difference in BCMA expression of MM patients in different types and stages (P>0.05), but it was found that for the newly diagnosed MM patients, the BCMA expression level of IgG patients was higher than that of IgA or light-chain patients (rank average 11.20 *vs* 5.44, P=0.014). There was no significant correlation between the BCMA expression and the age and serum creatinine of MM patients (P>0.05). And there was no significant difference in BCMA expression between patients with different levels of age and serum creatinine (P>0.05). But it was found that the BCMA expression level of the newly diagnosed MM patients was moderately positively correlated with their age (P=0.025, r=0.595). There was no significant correlation between the BCMA expression and serum β2-microglobulin, serum lactate dehydrogenase, free kap/lam ratio, and urine β2-microglobulin (P>0.05). But we found that the BCMA expression of patients with high serum β2-microglobulin was higher than that of patients with low serum β2-microglobulin (rank average 28.89 *vs* 17.54, P=0.017). And the BCMA expression of patients with abnormal serum free kap/lam ratio was higher than that of patients with normal ratio (rank average 28.49 *vs* 13.55, P=0.004). The BCMA expression was strongly positively correlated with 24-h urine protein, was moderately positively correlated with serum M protein and the percentage of plasma cells in bone marrow, was moderately negatively correlated with albumin and hemoglobin count, and was weakly positively correlated with serum corrected calcium (P<0.05). And it was found that the BCMA expression of positive serum immunofixation electrophoresis patients was higher than that of negative patients (rank average 29.94 *vs* 16.75, P=0.017). And we try to clarify the relationship between the bone marrow BCMA expression and the peripheral blood sBCMA expression. However, we have not found a clear correlation between them so far (P>0.05).

## Introduction

Multiple myeloma (MM) is a plasma cell malignant clonal proliferative tumor with high heterogeneity in natural clinical course (1). With the development of hematopoietic stem cell transplantation (HSCT) and the use of new drugs such as proteasome inhibitors, immunomodulators, monoclonal antibodies, and immune checkpoint inhibitors, the overall survival (OS) of MM has been significantly improved (2–5). However, because of the non-specific clinical manifestations, genetic instability, complex bone marrow microenvironment, and the recurrence and drug resistance of the disease, MM is still incurable at present (6–8).

In recent years, the chimeric antigen receptor T cell (CAR-T) targeting B cell maturation antigen (BCMA) immunotherapy clinical trials have achieved encouraging results, which brings hope to the cure of MM (6, 9, 10). BCMA, CD269, or the tumor necrosis factor receptor superfamily member 17 (TNFRSF17) is a kind of transmembrane glycoprotein that plays an important role in the differentiation of B cells and the proliferation of malignant myeloma cells by combining with BAFF and APRIL (11). BCMA is only expressed on late memory B cells and plasma cells, not on hematopoietic stem cells, progenitor cells, and non-hematopoietic organs (12). The expression level of BCMA on malignant plasma cells is generally higher than that on normal plasma cells (13). And Friedman et al. found that the expression level of BCMA on malignant plasma cells of MM patients is heterogeneous (14). Because of its restricted expression pattern and physiological function, BCMA is really an ideal target for MM CAR-T therapy (15, 16).

However, there are still some problems in the clinical trials of this immunotherapy, which are closely related to the expression of BCMA. Cytokine release syndrome (CRS) and neurotoxicity are common therapeutic toxic reactions in BCMA CAR-T clinical trials, which are related to the CAR-T cells’ activation and proliferation caused by the combination with BCMA (17). Relapse is also a problem that needs to be solved. Only 8–39% of the patients after therapy could achieve sustained very good partial response (VGPR) or complete response (CR), and many patients have different degrees of relapse (18). One of the reasons of relapse is the potential growth advantage of the original or new BCMA low expressed or BCMA^−^ malignant cells after treatment. And the relapse cases with BCMA low expressed or BCMA^−^ malignant cells have been found in the clinical trials of Brudno and Cohen et al. (19, 20). What’s more, the extracellular part of BCMA can be cut by the γ-secretase complex, and then the soluble B cell mature antigen (sBCMA) is released, which can affect the efficiency of the recognization or combination between CAR-T cells and BCMA and affect the effect of the therapy potentially (21, 22). So the expression level of BCMA is related to the safety and effect of the BCMA CAR-T therapy (18). However, the concrete relationship is still unclear. One clinical trial (No. ChiCTR-OPC-16009113) found that there was a significant difference in the OS and median progression-free survival (PFS) between patients with strong BCMA expression (>50%) and patients with low expression (<50%) (23). And it should be noted that the BCMA expression baseline requirements of various clinical trials are not uniform and highly heterogeneous at present, which has a direct impact on the size of the target patients of the BCMA CAR-T therapy (24).

In conclusion, it is of great significance for the optimization and promotion of MM BCMA CAR-T therapy to study the BCMA expression. In this study, 54 MM patients admitted to the Department of Hematology of the First Affiliated Hospital of Xi’an Jiaotong University from July 29, 2019, to October 31, 2019, were collected. The expression level of their bone marrow BCMA was detected by flow cytometry. The purpose was to determine the relationship between the expression of bone marrow BCMA and the classification, stage, and prognostic factors of MM, so as to identify the main factors that should be considered in the selection process of suitable MM patients for BCMA CAR-T therapy, and to provide guidance for the clinical application of this immunotherapy.

## Patients and Methods

### Patients and Data Collection

Our objective was to determine the relationship between the expression of bone marrow BCMA and the classification, stage, and prognostic factors of MM. A total of 54 MM patients treated in the Department of Hematology of the First Affiliated Hospital of Xi’an Jiaotong University from July 29, 2019, to October 31, 2019, were recruited in this study. Diagnosis assessment of the patients was performed according to the criteria of the guidelines for the diagnosis and management of multiple myeloma in China (2017 and 2020 revision) (25, 26). And patients with hemophilia, active infection, or other systemic tumor diseases and those in pregnancy or lactation were excluded. The expression level of their bone marrow BCMA was detected by flow cytometry, and data about their classification, stage, and prognostic factors were collected from the electronic medical records. This study has been registered in Chinese Clinical Trial Registry (ChiCTR1900025041) and was approved by the Ethics Committee of the First Affiliated Hospital of Xi’an Jiaotong University (XJTU1AF 2018LSK-025).

### Samples and Flow Cytometry Analysis

Bone marrow samples were obtained during the patients’ hospitalization and were used for flow cytometry analysis performed in a flow cytometer (Becton Dickinson, China) to assess the expression of BCMA. Combinations of four monoclonal antibodies were used (PE CD269/APCCD138/PERCP CD45/FITC CD38), and a minimum of 100,000 events were analyzed to determine the percentage of viable BCMA^+^ cells.

### Definition Criteria

The patients’ classification and stage of MM were defined following the definition of the guidelines for the diagnosis and management of multiple myeloma in China (2017, 2020 revision) (25, 26). In the analysis from the perspective of curative effect, the patients were divided into four groups: the newly diagnosed group, stable group, remission group, and relapse group. The evaluation of curative effect also referred to the guidelines mentioned above (25, 26). The stable group was determined according to the criterion of stable disease (SD) in the guideline. And the remission group included patients who met the standard of strict complete response (sCR), complete response (CR), very good partial response (VGPR), partial response (PR), or minute response (MR). The relapse group included progressive disease (PD), clinical relapse, and relapse from complete response of the guideline.

### Statistical Analyses

IBM SPSS Statistics software (version 24.0) was used for statistical analysis of the data. Demographic and clinical characteristics of all patients were summarized with descriptive statistics. The comparison of quantitative variables with uneven variance or non-normal distribution was assessed by non-parametric test. Two groups of variables were analyzed by Mann-Whiney U test, and three groups were tested by Kruskal-Wallis H test. Associations between two quantitative variables, at least one of which was non-normal distribution, were determined with Spearman correlation analysis. All tests were two-sided, and the value of P less than 0.05 was considered statistically significant.

## Results

In this study, a total of 54 MM patients were analyzed. The characteristics of the study patients are summarized in **Table 1**. The percentage of viable BCMA^+^ cells in bone marrow of each patient was detected by flow cytometry. The BCMA expression of the 54 MM patients was non-normal distribution, ranging from 0.000 to 24.510%, median 0.495%, interquartile range 2.847%, which is shown in the **Supplementary Table**. According to the curative effect, we classified the patients into four subgroups: the newly diagnosed group, stable group, remission group, and relapse group. The expression level of bone marrow BCMA in four different curative effect groups was statistically different (P=0.016). Though the difference in each pair of curative effect status was not statistically significant in the further paired analysis (P>0.05), it was found that the bone marrow BCMA expression level of the newly diagnosed group and relapse group was higher than that of the stable group and remission group (rank average 35.43 *vs* 21.61, P=0.001). We analyzed the expression of BCMA from different perspectives to determine the relationship between the bone marrow BCMA expression and the classification, stage, and prognostic factors of MM.

**Table 1:** Characteristics of the 54 patients.

Parameter	Number (%)
Sex	
Male	34 (63.0)
Female	20 (37.0)
Age (years)	
≥60	31 (57.4)
<60	23 (42.6)
Classification	
IgA	16 (29.6)
IgG	23 (42.6)
Light chain	13 (24.1)
IgM	1 (1.9)
IgD	1 (1.9)
ISS stage	
I	9 (16.7)
II	17 (31.5)
III	28 (51.9)
Subgroup	
Newly diagnosed	14 (25.9)
Stable	9 (16.7)
Remission	13 (24.1)
Relapse	18 (33.3)

### Analysis From the Perspective of Disease Classification

There was no significant difference in bone marrow BCMA expression level of 54 MM patients between IgG group, IgA group, light chain group, and other group (IgM, IgD), neither between IgA group and non-IgA group (P>0.05). We made further comparison analysis of the BCMA expression among different types of patients with different therapeutic states. There was no significant difference in the stable group, the remission group, and the relapse group (P>0.05). However, the bone marrow BCMA expression of patients with different disease types (IgG, IgA, and light chain) within the newly diagnosed group was different (P=0.048). Though the difference in each pair of disease type was not statistically significant in the further paired analysis of the newly diagnosed patients (P>0.05), it was found that the bone marrow BCMA expression level of the IgG newly diagnosed patients was higher than that of the IgA and light chain newly diagnosed patients (rank average 11.20 *vs* 5.44, P=0.014).

### Analysis From the Perspective of Disease Stage

There was no significant difference in bone marrow BCMA expression level of 54 multiple myeloma patients between ISS-I group, ISS-II group, and ISS-III group (P>0.05). And the difference among patients with different disease stages within different curative effect groups was also not statistically significant (P>0.05).

### Analysis From the Perspective of Prognostic Factors

We analyzed the correlation between the bone marrow BCMA expression and several MM prognostic factors, and compared the expression of BCMA in patients with different levels or types of each prognostic factor. The results are summarized in **Tables 2** and **3**, respectively.

**Table 2:** Correlation analysis of bone marrow BCMA expression and prognostic factors.

Prognostic factors	Spearman correlation coefficient (r)	P
Age	0.174	0.208
Hemoglobin	−0.406	0.002^*^
Serum creatinine	0.120	0.387
Serum corrected calcium	0.378	0.005^*^
24-h urine protein	0.606	0.006^*^
Albumin	−0.421	0.002^*^
Serum M protein	0.539	0.000^*^
Serum β2-MG	0.207	0.172
Urine β2-MG	0.135	0.338
LDH	−0.159	0.251
Free kap/lam ratio	0.231	0.097
Percentage of plasma cells in BM	0.471	0.000^*^

β2-MG, β2-microglobulin; LDH, lactate dehydrogenase; BM, bone marrow; *P < 0.05.

**Table 3:** Comparison of BCMA in patients with different levels/types of each prognostic factor.

Prognostic factors	Levels/types	Rank average of Mann-Whiney U test	P
Age	<60 years	24.85	0.286
	≥60 years	29.47	
Sex	Male	27.65	0.929
	Female	27.25	
Serum creatinine	<177 umol/L	26.71	0.492
	≥177 umol/L	30.25	
Albumin	<35 g/L	35.65	0.003^*^
	≥35 g/L	22.71	
Serum β2-MG	<2,300 ug/L	17.54	0.017^*^
	≥2,300 ug/L	28.89	
Serum M protein	<20%	22.83	0.006^*^
	≥20%	34.83	
Serum immunofixation electrophoresis	Negative	16.75	0.017^*^
	Positive	29.94	
LDH	<250 U/L	25.58	0.665
	≥250 U/L	27.94	
Free kap/lam ratio	Normal	13.55	0.004^*^
	Abnormal	28.49	
Percentage of plasma cells in BM	<30%	21.24	0.002^*^
	≥30%	34.76	
P53 deletion	Yes	22.13	0.787
	No	23.32	
IGH translocation	Yes	24.50	0.433
	No	21.43	
RB1 deletion	Yes	21.72	0.594
	No	23.85	
1q21 amplification	Yes	23.48	0.774
	No	22.34	
D13S319 deletion	Yes	18.56	0.092
	No	25.45	

β2-MG, β2-microglobulin; LDH, lactate dehydrogenase; BM, bone marrow; *P < 0.05.

Though there was no significant correlation between the bone marrow BCMA expression and patients’ age (P>0.05), it was found that the bone marrow BCMA expression level of the newly diagnosed patients was moderately positively correlated with their age (P=0.025, r=0.595).The results showed that the bone marrow BCMA expression was strongly positively correlated with 24-h urine protein, was moderately positively correlated with serum M protein and the percentage of plasma cells in bone marrow, was moderately negatively correlated with albumin and hemoglobin count, and was weakly positively correlated with serum corrected calcium (P<0.05). It was found that the bone marrow BCMA expression of positive serum immunofixation electrophoresis patients was higher than that of negative patients (rank average 29.94 *vs* 16.75, P=0.017). The patients with high serum β2-microglobulin (β2-MG) had higher bone marrow BCMA expression than the patients with low serum β2-MG (rank average 28.89 *vs* 17.54, P=0.017). And the bone marrow BCMA expression of patients with abnormal serum free kap/lam ratio was higher than that of patients with normal ratio (rank average 28.49 *vs* 13.55, P=0.004).

### Analysis of sBCMA

We also did some research on sBCAM. Up to now, we have detected the expression level of BCMA in peripheral blood of 97 MM patients by using the human BCMA/TNFRSF17 DuoSet ELISA kit (R&D) and made statistical analysis on 38 of them, which have detected the expression level of BCMA in their bone marrow at the same time. However, we have not found a clear correlation between them so far (P>0.05).

## Discussion

### The Significance of Studying BCMA Expression

MM is a plasma cell malignant clonal proliferative tumor. In recent years, the treatment of MM has made great progress. The overall survival (OS) of MM has been significantly improved with the development of new drugs and new strategies (27–30). However, MM still remains refractory and incurable so far (6). It is worth noting that CAR-T immunotherapy has offered encouraging results in the clinical trials of MM. The appropriate target is the key to success in this therapy.

At present, many targets of MM CAR-T therapy are being studied, such as BCMA, kappa light chain, SLAMF7, GPRC5D, activated integrin β7, CD19, CD138, etc. (15). BCMA is a hot topic of the current research. BCMA is only expressed on late memory B cells and plasma cells, which is really a restricted expression pattern. And it plays an important role in the differentiation of B cells and the proliferation of malignant myeloma cells. Furthermore, the expression level of BCMA in malignant plasma cells is generally higher than that in normal plasma cells, and its downregulation can inhibit the proliferation of multiple myeloma cells (31). All these characteristics make BCMA an ideal target for MM CAR-T immunotherapy.

Though the clinical trials of BCMA CAR-T therapy have achieved encouraging results, which brings hope to the cure of MM (9, 10, 19), some problems prevent its further promotion in clinical practice. As mentioned at the beginning of the article, the problems need to be solved in the trials such as therapeutic toxic reactions, clinical relapse, and patients’ inclusion criteria are closely related to the expression of BCMA. And Friedman et al. found that the expression level of BCMA in malignant plasma cells of MM patients is heterogeneous (14). However, study of the BCMA expression remains limited so far.

Our team studied some clinical trials of the BCMA CAR-T therapy and found that the BCMA expression baseline requirements of various trials were not uniform and highly heterogeneous (9). To date, no consensus about the patients’ inclusion criteria for the therapy has been systematically achieved. One study has shown that high requirements for BCMA positive rates can prevent about one-third of MM patients from receiving the BCMA CAR-T therapy (24). So it is of great significance to study the BCMA expression, and we hope the results of our study can provide reference for the clinical application of this immunotherapy.

We report here the results of a study on the relationship between the expression of bone marrow BCMA and the classification, stage, and prognostic factors of MM, hoping to identify the main clinical factors that should be considered in the selection of suitable MM patients for BCMA CAR-T therapy.

### From the Perspective of Curative Effect

It is worth noting that the expression level of bone marrow BCMA in four different curative effect groups was statistically different in our study (P=0.016). Though there was no statistical result in the further paired analysis (P>0.05), it was found that the BCMA expression of the newly diagnosed group and relapse group was higher than that of the stable group and remission group (rank average 35.43 *vs* 21.61, P=0.001), which indicates that patients’ curative effect may be a factor that needs to be considered in the selection of appropriate patients for BCMA CAR-T therapy. It can be assumed that the difference of BCMA expression among MM patients with different curative effect may be more obvious with the increase of sample size. Further study needs to be done to clarify the significance of curative effect for the patient selection of BCMA CAR-T therapy.

### From the Perspective of Disease Classification

The results of our study showed that there was no significant difference in bone marrow BCMA expression of MM patients with different types (P>0.05), which indicates that patients’ MM classification may not be the main factor that needs to be considered in the selection of suitable patients for BCMA CAR-T therapy. But we found that for the newly diagnosed MM patients, the bone marrow BCMA expression of patients with different disease types was different (P=0.048). Though there was no statistical result in the further paired analysis, it was found that the bone marrow BCMA expression of the IgG newly diagnosed patients was higher than that of IgA and light chain patients (rank average 11.20 *vs* 5.44, P=0.014). It can be assumed that the difference of BCMA expression among different types of the newly diagnosed MM patients may be more obvious with the increase of sample size. It is worth doing further study to clarify the significance of disease classification for the newly diagnosed MM patients in the selection of BCMA CAR-T therapy.

### From the Perspective of Disease Stage

The difference of bone marrow BCMA expression among patients with different disease stages was not statistically significant (P>0.05), which indicates that patients’ ISS stage may not be the main factor that needs to be considered in the selection of suitable patients for BCMA CAR-T therapy.

### From the Perspective of Prognostic Factors

We found some factors that were related to the bone marrow BCMA expression, which seem to make sense on the base of the existing knowledge of those factors and BCMA.

The results of a multivariate survival analysis performed by Kyle Robert A et al. on 1,027 MM patients showed that age, proportion of plasma cells, platelet count, serum albumin, and serum creatinine were the most important prognostic factors for multiple myeloma (32). In our study, it was found that the bone marrow BCMA expression level of the newly diagnosed patients was moderately positively correlated with their age (P=0.025, r=0.595), which indicates that the age may be a factor that needs to be considered in the selection of BCMA CAR-T therapy for the newly diagnosed MM patients. And the results showed that the BCMA expression of MM patients was moderately positively correlated with the percentage of plasma cells in their bone marrow (P=0.000, r=0.471), which is in line with clinical practice. But we did not find significant correlation between the bone marrow BCMA expression and the serum creatinine level (P>0.05). Studies have shown that there is a negative correlation between the serum albumin level of MM patients and the expression of IL-6, which is related to the abnormal proliferation of myeloma cells, so the serum albumin level can be used as an important serological marker to evaluate the tumor load of MM (33). And it has been confirmed that the expression level of BCMA in malignant plasma cells is generally higher than that in normal plasma cells. In our study, we found the BCMA expression level was moderately negatively correlated with albumin count, which is in line with the results of the studies mentioned above.

Anemia of MM patients is related to the myelosuppression caused by the malignant proliferation and infiltration of myeloma cells, so the severity of anemia can indirectly reflect the tumor load and affect the prognosis of MM. We found that the bone marrow BCMA expression was moderately negatively correlated with hemoglobin count (P=0.002, r=−0.406), which is consistent with clinical practice. Serum β2-MG is secreted by plasma cells and is positively correlated with the total number of myeloma cells. We did not find significant correlation between the bone marrow BCMA expression and the patients’ serum β2-MG (P>0.05), but results showed that the MM patients with high serum β2-MG had higher bone marrow BCMA expression than the patients with low serum β2-MG (rank average 28.89 *vs* 17.54, P=0.017), which makes sense. Serum lactate dehydrogenase (LDH), an important metabolic enzyme produced in the process of glycolysis, is closely related to the disease activity and tumor load of MM, which has an important reference value in the prognosis evaluation of multiple myeloma patients. However, the results of our study did not show significant correlation between the bone marrow BCMA expression and the patients’ serum LDH (P>0.05).

Studies have shown that the increase of serum free light chain (sFLC) level accompanied by abnormal kap/lam ratio can reflect the abnormal expansion of clonal plasma cells and is positively related to the poor prognosis of MM (34). In our study, there was no significant correlation between the bone marrow BCMA expression and serum free kap/lam ratio (P>0.05), but we found that the BCMA expression level of MM patients with abnormal serum free kap/lam ratio was higher than that of patients with normal ratio (rank average 28.49 *vs* 13.55, P=0.004), which seems to be supported by the conclusions of the study mentioned above.

More than 90% of MM patients have cytogenetic abnormalities that are related to disease recurrence and prognosis (35–38). We try to analyze the relationship between the bone marrow BCMA expression and some gene mutations including P53 deletion, IGH translocation, RB1 deletion, 1q21 amplification, and D13S319 deletion, but the results were not statistically significant (P>0.05). One study has demonstrated that early monoclonal protein (M protein) decline pattern is an independent prognostic factor in patients with multiple myeloma (39), and we found that the bone marrow BCMA expression was moderately positively correlated with serum M protein (P=0.000, r=0.539). Furthermore, we found that the BCMA expression of positive serum immunofixation electrophoresis MM patients was higher than that of negative patients (rank average 29.94 *vs* 16.75, P=0.017), and MM patients’ bone marrow BCMA expression was strongly positively correlated with their 24-h urine protein (P=0.006, r=0.606) and was weakly positively correlated with serum corrected calcium (P=0.005, r=0.378), which can provide reference for the evaluation of bone marrow BCMA expression of MM patients in clinical practice.

In conclusion, according to our results, prognostic factors including age (for the newly diagnosed patients), 24-h urine protein, serum M protein, the percentage of plasma cells in bone marrow, albumin, hemoglobin, serum corrected calcium, serum β2-MG, serum free kap/lam ratio, and serum immunofixation electrophoresis may need to be considered in the selection of suitable patients for BCMA CAR-T therapy. And what’s more, these factors can be used to make a rough estimate of the bone marrow BCMA expression of MM patients in clinical practice, which is of great significance to save clinical cost.

## Limitations and Prospects

There are several limitations in our study, including very small sample size, no controls, and the inherent defects in a single-center study. A multicenter study with larger sample size is warranted. And in our study, we detected the percentage of viable BCMA^+^ cells in bone marrow of each MM patient by flow cytometry to reflect the expression level of BCMA, which cannot fully represent its expression intensity. It is worth doing further study to clarify the relationship between the bone marrow BCMA expression and the peripheral blood sBCMA expression, which is easier to obtain in clinical practice. Our team has been doing relevant research at present. However, we have not found a clear correlation between them so far. Also, it makes sense to explore the relationship between sBCMA and the classification, stage, and prognostic factors of MM. We will expand the sample size to do further exploration, hoping to provide clinically significant results for the promotion of the BCMA CAR-T therapy.

## Data Availability Statement

The raw data supporting the conclusions of this article will be made available by the authors, without undue reservation.

## Ethics Statement

The studies involving human participants were reviewed and approved by the Ethics Committee of the First Affiliated Hospital of Xi’an Jiaotong University. The patients/participants provided their written informed consent to participate in this study.

## Author Contributions

Conception and design: HL. Design implementation: TM. Managed the patient information and contributed to data acquisition: HL, MW. Data collecting: TM, JS, YX, and TB. Samples detecting: TM. Assisted in samples detecting: LH and JW. Statistical analysis and manuscript writing: TM. Revised the present manuscript: HL. All authors contributed to the article and approved the submitted version.

## Funding

This study received funding from EUREKA BJ Therapeutics, Inc, the Shaanxi Province Science and Technology research Projects (Grant No.2016SF-122) and The Provincial University Joint Project - General Project (Grant No.2020GXLH-Y-006). The funders were not involved in the study design, collection, analysis, interpretation of data, the writing of this article, or the decision to submit it for publication.

## Conflict of Interest

The authors declare that the research was conducted in the absence of any commercial or financial relationships that could be construed as a potential conflict of interest.

## Publisher’s Note

All claims expressed in this article are solely those of the authors and do not necessarily represent those of their affiliated organizations, or those of the publisher, the editors and the reviewers. Any product that may be evaluated in this article, or claim that may be made by its manufacturer, is not guaranteed or endorsed by the publisher.
