# Supplementary material for: Study on the Relationship Between the Expression of B Cell Mature Antigen and the Classification, Stage, and Prognostic Factors of Multiple Myeloma
Source: Front Immunol. 2021 Nov 18;12:724411. doi: 10.3389/fimmu.2021.724411 (PMC8637449; doi:10.3389/fimmu.2021.724411)
Supplement: Supplementary file 1 [file Table_1.docx]

**Supplementary table** BCMA expression in bone marrow of the 54 MM patients

| Patients | Sex | Age (years) | Classification | ISS stage | Group | BCMA(%) |
| --- | --- | --- | --- | --- | --- | --- |
| 1 | M | 46 | Light chain | Ⅲ | 3 | 0.067 |
| 2 | M | 46 | Light chain | Ⅲ | 3 | 0.417 |
| 3 | M | 69 | IgA | Ⅲ | 4 | 0.012 |
| 4 | M | 43 | Light chain | Ⅲ | 4 | 0.173 |
| 5 | M | 43 | Light chain | Ⅲ | 4 | 0.030 |
| 6 | F | 64 | Light chain | Ⅲ | 3 | 0.070 |
| 7 | M | 51 | IgA | Ⅰ | 3 | 0.027 |
| 8 | F | 68 | IgG | Ⅰ | 4 | 0.384 |
| 9 | M | 69 | IgG | Ⅱ | 4 | 0.000 |
| 10 | M | 62 | IgG | Ⅱ | 3 | 0.194 |
| 11 | F | 66 | IgG | Ⅲ | 4 | 0.303 |
| 12 | F | 60 | IgG | Ⅱ | 3 | 0.008 |
| 13 | M | 50 | IgG | Ⅲ | 3 | 0.022 |
| 14 | M | 69 | IgG | Ⅰ | 4 | 0.000 |
| 15 | F | 70 | IgG | Ⅲ | 1 | 2.442 |
| 16 | M | 63 | IgG | Ⅱ | 1 | 10.638 |
| 17 | M | 71 | IgA | Ⅲ | 1 | 2.857 |
| 18 | F | 67 | IgG | Ⅱ | 2 | 0.970 |
| 19 | F | 58 | IgA | Ⅰ | 1 | 0.405 |
| 20 | F | 58 | IgA | Ⅰ | 4 | 3.229 |
| 21 | F | 64 | IgG | Ⅱ | 3 | 24.510 |
| 22 | M | 75 | IgA | Ⅲ | 3 | 0.163 |
| 23 | M | 46 | IgG | Ⅱ | 4 | 9.355 |
| 24 | M | 46 | IgG | Ⅱ | 4 | 0.130 |
| 25 | F | 81 | IgG | Ⅱ | 1 | 2.792 |
| 26 | F | 81 | IgG | Ⅱ | 4 | 0.080 |
| 27 | M | 29 | IgG | Ⅱ | 4 | 1.050 |
| 28 | M | 48 | Light chain | Ⅲ | 4 | 0.036 |
| 29 | F | 56 | Light chain | Ⅲ | 1 | 0.170 |
| 30 | M | 57 | Light chain | Ⅲ | 4 | 0.706 |
| 31 | M | 67 | IgG | Ⅲ | 2 | 23.015 |
| 32 | M | 48 | IgG | Ⅱ | 2 | 4.897 |
| 33 | F | 53 | IgA | Ⅲ | 2 | 0.573 |
| 34 | M | 55 | Light chain | Ⅲ | 2 | 22.880 |
| 35 | M | 32 | IgD | Ⅲ | 4 | 4.963 |
| 36 | M | 73 | IgA | Ⅲ | 2 | 12.536 |
| 37 | F | 73 | IgA | Ⅱ | 1 | 1.614 |
| 38 | M | 74 | IgG | Ⅲ | 1 | 15.331 |
| 39 | M | 63 | IgA | Ⅲ | 1 | 2.159 |
| 40 | M | 64 | IgA | Ⅲ | 1 | 0.000 |
| 41 | M | 68 | IgG | Ⅲ | 3 | 0.163 |
| 42 | F | 74 | IgG | Ⅰ | 2 | 0.820 |
| 43 | F | 73 | IgA | Ⅱ | 3 | 3.126 |
| 44 | F | 47 | IgA | Ⅱ | 4 | 0.146 |
| 45 | M | 50 | Light chain | Ⅱ | 1 | 1.192 |
| 46 | M | 62 | Light chain | Ⅰ | 1 | 0.407 |
| 47 | M | 75 | IgA | Ⅱ | 4 | 2.733 |
| 48 | M | 73 | Light chain | Ⅲ | 1 | 8.119 |
| 49 | F | 53 | IgM | Ⅰ | 3 | 1.106 |
| 50 | M | 57 | IgA | Ⅲ | 2 | 0.000 |
| 51 | M | 75 | IgA | Ⅲ | 2 | 0.663 |
| 52 | M | 66 | IgG | Ⅲ | 1 | 9.874 |
| 53 | F | 68 | Light chain | Ⅲ | 4 | 0.371 |
| 54 | F | 56 | IgG | Ⅰ | 3 | 0.009 |

*F* female, *M* male,*Group1* the newly diagnosed group, *Group2* stable group,*Group3* remission group, *Group4* relapse group
